# Irinotecan-Loaded Polymeric Micelles as a Promising Alternative to Enhance Antitumor Efficacy in Colorectal Cancer Therapy

**DOI:** 10.3390/polym14224905

**Published:** 2022-11-14

**Authors:** Fernanda Lapa Campos, Janaina de Alcântara Lemos, Caroline Mari Ramos Oda, Juliana de Oliveira Silva, Renata Salgado Fernandes, Sued Eustaquio Mendes Miranda, Carolina Henriques Cavalcante, Geovanni Dantas Cassali, Danyelle M. Townsend, Elaine Amaral Leite, Andre Luis Branco de Barros

**Affiliations:** 1 Department of Pharmaceutical Products, Faculty of Pharmacy, Federal University of Minas Gerais, Belo Horizonte 31270-901, Brazil; 2 Department of General Pathology, Institute of Biological Sciences, Federal University of Minas Gerais, Belo Horizonte 31270-901, Brazil; 3 Department of Drug Discovery and Pharmaceutical Sciences, Medical University of South Carolina, Charleston, SC 29425, USA; 4 Department of Clinical and Toxicological Analyses, Faculty of Pharmacy, Federal University of Minas Gerais, Belo Horizonte 31270-901, Brazil

**Keywords:** micelles, irinotecan, colorectal cancer, drug delivery, antitumor activity

## Abstract

Colorectal cancer has been considered a worldwide public health problem since current treatments are often ineffective. Irinotecan is a frontline chemotherapeutic agent that has dose-limiting side effects that compromise its therapeutic potential. Therefore, it is necessary to develop a novel, targeted drug delivery system with high therapeutic efficacy and an improved safety profile. Here, micellar formulations composed of 1,2-distearoyl-sn-glycero-3-phosphoethanolamine-N-[methoxy(polyethyleneglycol)-2000] (DSPE-mPEG_2k_) containing irinotecan were proposed as a strategy for colorectal cancer therapy. Firstly, the irinotecan-loaded micelles were prepared using the solvent evaporation method. Then, micelles were characterized in terms of size, polydispersity, zeta potential, entrapment efficiency, and release kinetics. Cytotoxicity and in vivo antitumor activity were evaluated. The micelles showed size around 13 nm, zeta potential near neutral (−0.5 mV), and encapsulation efficiency around 68.5% (irinotecan 3 mg/mL) with a sustained drug release within the first 8 h. The micelles were evaluated in a CT26 tumor animal model showing inhibition of tumor growth (89%) higher than free drug (68.7%). Body weight variation, hemolytic activity, hematological, and biochemical data showed that, at the dose of 7.5 mg/kg, the irinotecan-loaded micelles have low toxicity. In summary, our findings provide evidence that DSPE-mPEG_2k_ micelles could be considered potential carriers for future irinotecan delivery and their possible therapeutic application against colorectal cancer.

## 1. Introduction

Cancer is a disease that threatens global health, and the number of new cases and deaths increases annually. Colorectal cancer (CRC) accounts for 10% of the global incidence and is the second leading cause of cancer-related death worldwide [1]. Among the antineoplastic agents available for CRC treatment, irinotecan (IRN), a topoisomerase I inhibitor, is used in monotherapy or combined with 5-fluorouracil, oxaliplatin, and leucovorin [2,3]. Nevertheless, the clinical use of the IRN has been limited by fast clearance from the blood, systemic toxicity, and low tolerability in patients [4].

There have been enormous advancements in the area of drug delivery systems to overcome bioavailability and off target toxicity, particularly in the cancer drug development arena. One such success is Onivyde, a liposomal formulation of irinotecan, approved by the US Food and Drug Administration (FDA) as a second-line treatment for metastatic pancreatic adenocarcinoma [3,5]. This formulation has more favorable pharmacokinetics with an enhanced time in circulation; however, neutropenia persists as a clinical problem. Given these restrictions, there remains an unmet need for developing new drug delivery systems capable of improving therapeutic efficacy and reducing side effects [5].

Micelles are nanocarriers that may be promising drug delivery systems, particularly for lipophilic drugs. Among the available nanocarriers, polymeric micelles are spherical colloidal particles composed of amphiphilic molecules, which, when reaching the critical micelle concentration (CMC), form a “shell-core” structure spontaneously assembled and dispersed in water [6,7]. The application of micelles as drug nanocarriers presents some advantages such as solubilization of poorly water-soluble substances, protection of drugs against degradation reactions, changes in biodistribution, decreased unwanted side effects, and increased maximum tolerated dose [8].

Polymer micelles are composed of block copolymers or lipids associated with polymers, which are hydrophobic lipidic chains linked to hydrophilic polymeric chains. Hydrophobic fragments of the block copolymer form the micelle’s core while the hydrophilic fragments make up the shell. Different polymers can be used to form micelles; nevertheless, the selection is limited to biocompatible and biodegradable materials. The hydrophilic shell is usually composed of polyethylene glycol (PEG) as it has low toxicity, is highly hydratable and electrically neutral, and besides avoids nanoparticle aggregation. It also reduces interactions between blood plasma components and nanoparticles which prevents clearance by the mononuclear phagocytic system (MPS) and results in longer blood circulation time and potentially tumor accumulation [8,9,10,11]. In turn, the lipophilic part of the micelles can be composed of polymers or lipids, such as 1,2-distearoyl phosphatidylethanolamine (DSPE), selected according to the nature of the drug to be encapsulated [12]. Several studies using DSPE-PEG-based micelle have shown the ability of this nanocarrier to achieve high payloads for hydrophobic or amphiphilic antitumor drugs [13,14,15,16], accumulate in tumors due to its small particle size [17,18], and reduce the toxicity of drugs [19].

Based on the above, we leveraged this approach for in the design and development of a systemic delivery of IRN into DSPE-PEG-based polymeric micelles to improve the efficacy and mitigate possible systemic toxic effects in cancer chemotherapy. As a proof-of-concept study, we took advantage of the small size of polymeric micelles and great compatibility to PEG coating to effectively prepare an IRN delivery system with favorable characteristics for in vivo antitumor application. Therefore, in this study, we described the preparation and characterization of IRN-loaded polymeric micelles, drug release profile study, and hemocompatibility assay. Next, we investigated in vivo antitumor effect and treatment toxicity of the proposed micellar formulation as a promising alternative for CRC therapy.

## 2. Materials and Methods

Trihydrated irinotecan hydrochloride (IRN) was donated from EUROFARMA Laboratórios SA (São Paulo, Brazil). The 1,2-distearoyl-sn-glycero-3-phosphoethanolamine-N-[methoxy(polyethyleneglycol)-2000] (DSPE-mPEG_2k_) were supplied by Lipoid GmbH (Ludwigshafen, Germany). Triethylamine and chloroform were obtained from Sigma-Aldrich Chemical Company (St Louis, MO, USA). The HPLC-grade acetonitrile (ACN) and isopropyl alcohol were obtained from Tedia Company (Fairfield, OH, USA). Glucose was purchased from Vetec Química Fina Ltda (São Paulo, Brazil). The water was purified using Milli-Q^®^ equipment (Millipore, Burlington, MA, USA). All other solvents of the analytical grade and reagents were purchased from Sigma-Aldrich (São Paulo, Brazil).

### 2.1. Preparation of Micelles

The polymeric micelles were prepared as described previously by [18], using the solvent evaporation method, with some modifications. In brief, DSPE-mPEG2k (10 mmol·L^−1^) was dissolved in chloroform solution and then a rotary evaporator (Buchi Labortechnik AG CH-9233, model R-210) setup at 30 °C, 130 rpm, and 131 mbar pressure (V-700 vacuum pump, Flawil, Switzerland) was used to remove the organic solvent and form a thin film. Then, the thin film was hydrated with 0.9% (w/v) sodium chloride solution at 40 °C for 5 min, followed by vortexing with agitation (1000 rpm) to form blank micelles, named PM DSPE-PEG.

#### Irinotecan-Loaded Micelles

The incorporation of IRN into PM DSPE-PEG was performed as reported by [20] with some adjustments. Briefly, the PM DSPE-PEG were incubated with an IRN solution for 30 min at 60 °C to allow drug loading at 1 mg/mL concentration. After incubation, the micellar formulations were washed and purified three times using Amicon^®^ 30 kDa devices MWCO (Millipore, Burlington, MA, USA) by centrifugation at 10,000 rpm for 10 min (Heraeus Multifuge X1R-Centrifuge, Thermo Fisher Scientific, Waltham, MA, USA). The washed particles were resuspended in water, obtaining formulations named PM DSPE-PEG IRN.

### 2.2. Physicochemical Characterization

#### 2.2.1. Mean Diameter and Size Distribution

The mean diameter and the size distribution of the micelles were determined by Dynamic Light Scattering (DLS), at 25 °C, at an angle of 90°, using a Nano ZS 90 Zetasizer (Malvern Instruments, Worcestershire, UK). The samples were diluted using 0.9% (w/v) sodium chloride, at a proportion of 1:10 (v/v). Micelle’s mean diameter measurements were further performed by Small Angle X-Ray Scattering (SAXS) as previously described by our research group [14].

#### 2.2.2. Zeta Potential

The zeta potential was determined by DLS associated with electrophoretic mobility. All measurements were carried out in triplicate using a Nano ZS 90 Zetasizer (Malvern Instruments, England) [21]. To measure the zeta potential, the samples were prepared as outlined in Section 2.2.1.

#### 2.2.3. Entrapment Efficiency

The entrapment efficiency (EE) of IRN into micelles was determined by ultrafiltration-centrifugation, at 10,000× *g* for 10 min, at 25 °C, using 0.5 mL ultrafilter devices (MW cutoff 30 kDa, Millipore, Burlington, MA, USA). The concentration of IRN in the micelles before (non-purified micelles) and after centrifugation (purified micelles) was determined by an ultraviolet-visible (UV–Vis) spectrophotometer (Evolution 201 UV-Visible Spectrophotometer Thermo Scientific, Waltham, MA, USA) at 370 nm [22]. The percent entrapment efficiency (EE%) was calculated from the equation given below:

EE(%) = ([IRN]_purified micelles_/[IRN]_non-purified micelles_) × 100 (1)

### 2.3. Short-Term Stability Evaluation

The storage stability of micelles maintained at 4 °C was evaluated at 0, 1, 4, 7, and 15 days after preparation. The physicochemical characteristics explored were the mean diameter, size distribution, zeta potential, and EE [23]. For each time measured, the samples were purified as in Section 2.2.3. The mean values of the physicochemical characteristics evaluated were compared with date obtained at time zero.

### 2.4. Preparation of Freeze-Dried Micelles for IRN Encapsulation

PM DSPE-PEG were lyophilized using glucose as a cryoprotectant in the mass ratio of 2:1 (sugar to polymer). The blank micelles were lyophilized as described in previous reports [14,18]. The PM formulations were reconstituted by adding IRN solution at different concentrations (1, 2, and 3 mg/mL), and drug-loaded formulations were kept in a water bath at 60 °C for 30 min to optimize drug encapsulation. Then, the PM formulations were purified and characterized as previously described in Section 2.2.

### 2.5. In Vitro IRN Release Study

IRN release parameters was evaluated using the dialysis method in phosphate buffer (PBS, pH 7.4) [24]. Briefly, 0.4 mL of the IRN-loaded micelles or free IRN was dialyzed against 40 mL of the buffer and gently shaken at 150 rpm and 37 °C (IKA KS 4000i control, Campinas, Brazil). Aliquots of 1 mL of the external medium were withdrawn after predetermined time intervals (0.5, 1, 2, 4, 6, 8, and 24 h) with the replacement of 1 mL fresh PBS. The amount of IRN released from the micelles was measured by high-performance liquid chromatography (HPLC) as described in previous reports [25,26,27].

### 2.6. Hemolysis Assay

Hemolysis tests were conducted in vitro following a method previously published [28]. PM DSPE-PEG IRN dispersions (3 mg/mL), and IRN solution (free IRN) were added into 4% red blood cells in a 0.9% (w/w) aqueous NaCl solution at designated concentrations (200, 500, and 800 µg/mL). All the samples were incubated for 1 h at temperature (37 °C), and agitation (500 bpm) (metabolic bath, Dubnoff MA-95/CF Marconi, Piracicaba, SP, Brazil). Following incubation, the samples were centrifuged at 3000 rpm for 10 min and the absorbance of the supernatants was measured in a spectrophotometer (Evolution 201 UV–vis Spectrophotometer Thermo Scientific, Waltham, MA, USA) at 540 nm. The percentage of hemolysis was then calculated using the following equation:

%hemolysis = ((Abs_sample_ − Abs_negative control_)/(Abs_positive control_ − Abs_negative control_)) × 100 (2)

### 2.7. In Vivo Studies

#### 2.7.1. Animals

Female BALB/c mice (8–10-wk-old, 20 ± 2 g) were acquired from CEBIO-UFMG (Belo Horizonte, Brazil). Animals were kept under a controlled environment to a temperature range of 25 ± 2 °C and a humidity range of 30–70%, with a 12 h light–dark cycle, and free access to standard food and water. All animal studies were approved by the local Ethics Committee for Animal Experiments of the Federal University of Minas Gerais, Brazil (protocol # 311/2018, approval date 9 October 2018).

#### 2.7.2. Cell Culture

A murine colorectal cancer cell line (CT26) was used for tumor development. Cells were grown in RPMI-1640 medium with FBS (10% v/v), penicillin (1% w/v), and streptomycin (1% v/v) at 37 °C in a 5% CO_2_ atmosphere. After confluence, the cells were trypsinized and a culture medium suspension, at a concentration of 1.0 × 10^7^ CT26 cells/mL, was prepared for tumor inoculation. Then, an aliquot of 0.1 mL was subcutaneously injected into the right flank of each mouse. Antitumor study was initiated when tumor reached about 100 mm^3^.

#### 2.7.3. Antitumor Activity Evaluation

Mice were randomly divided into three groups (N = 6): (1) saline (control), (2) free IRN (IRN solution), and (3) PM DSPE-PEG IRN. The mice received, by the tail vein, every 2 days, a dose of 7.5 mg/kg of IRN with reference to previous reports [29]. The total number of administration was six, leading to a cumulative dose of 45 mg/kg. All the mice were euthanized two days after the last administration (D_12_). Body weight and tumor volume were recorded every 2 days. The tumor volume was calculated by the following equation:

V = (d_1_)^2^ × d_2_ × 0.5 (3)

where d_1_ and d_2_ represent the smaller and larger diameter, respectively. Relative tumor volume (RTV) and inhibition rate (IR) were calculated using the following formulas:

RTV = tumor volume on day 12/tumor volume on the first day of treatment(4)

IR = 1 − (Mean RTV of treatment group/Mean RTV of saline group) × 100(5)

#### 2.7.4. Histopathological Analysis

Tumors were harvested for histopathological analysis at the end of the study. The samples were fixed in 10% neutral formalin for 24 h, then dehydrated in alcohol and included in paraffin blocks, and cut into 5 μm-thick sections. Finally, the sections were stained with hematoxylin and eosin (H&E). After, the stained sections were visualized using an optical microscope (Olympus BX-40; Olympus, Tokyo, Japan).

#### 2.7.5. Toxicity Evaluation

During the in vivo antitumor activity experiment, mice were monitored according to their behavioral/clinical changes, body weight, and mice mortality. At the end of the study, blood was harvested by puncturing the brachial plexus from anesthetized mice and used for hematological and biochemical analysis. The hematological study included hemoglobin, number of red blood cells, hematocrit, hematometric indices, red blood cell distribution width (RDW), global and differential leukocyte count, and number of platelets as described previously [30].

Blood samples were centrifuged at 3000 rpm for 15 min, at 25 °C, to obtain the plasma for the renal (urea and creatinine) and hepatic (alanine aminotransferase—ALT and aspartate aminotransferase—AST) biochemical study. Measurements were carried out using commercial kits from Labtest^®^ (Lagoa Santa, Brazil) in the Bioplus BIO-2000 semiautomatic analyzer equipment (São Paulo, Brazil).

### 2.8. Statistical Analysis

Data were presented as mean ± standard deviation (SD). The statistical differences between the experimental groups were evaluated using GraphPad PRISM, version 8.00 software (GraphPad Software Inc., La Jolla, CA, USA). One-way analysis of variance (ANOVA), followed by the Tukey test, or T-test, when the number of groups evaluated was equal to two, were used for statistical testing. The normality and homogeneity of variance analyses were performed by D’Agostino–Pearson and Brown–Forsythe tests. Significant difference was considered for P-values lower than 0.05.

## 3. Results

### 3.1. Physicochemical Characterization

The physicochemical properties of the blank and drug-loaded micelles such as mean diameter, cumulative size distribution (D90), and zeta potential were determined by dynamic light scattering (DLS) and the results are summarized in Table 1.

**Figure 1:**
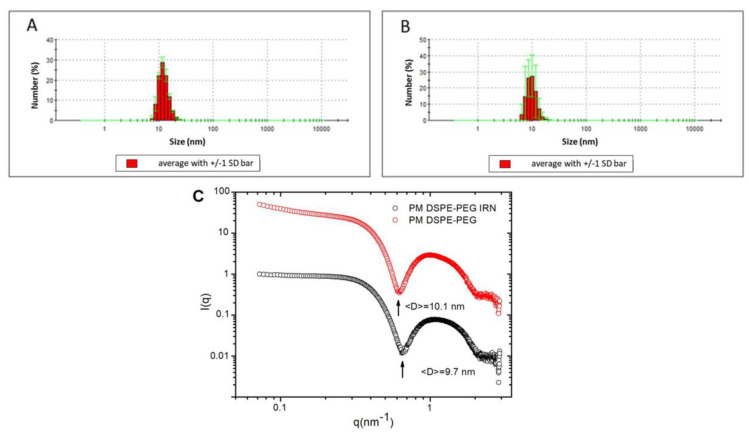
Size distribution for micellar formulations: (**A**) blank PM DSPE-PEG. Green bars represent SD of the mean values (**B**) PM DSPE-PEG IRN. Green bars represent SD of the mean values, and (**C**) SAXS pattern of PM DSPE-PEG and PM DSPE-PEG IRN. Black arrows correspond to the scattering vector condition (q).

IRN-loaded or blank micelles showed an average size of around 13 nm, while 90% of micelles (D90) showed a mean diameter smaller than 20 nm, which indicates homogeneous and uniform micellar systems (Figure 1A,B). The mean diameter was also estimated by SAXS. The measurement X-ray scattered intensities I(q) shown in Figure 1C for both formulations exhibited well-defined local minima that are compatible with the scattering profile from a mono-disperse micelle system. After 20 measures, the diameter was calculated using an equation proposed by [31], D = 2π/q, where “D” is the diameter of the micelles, and q (scattering vector condition) corresponds to the position of the first intensity minimum observed [I(q)] (indicated by the black arrows of Figure 1C). This analysis provides a mean diameter of 10.1 nm and 9.7 nm for PM DSPE-PEG and PM DSPE-PEG IRN, respectively. These findings are consistent with those obtained by DLS. Studies on micellar delivery systems have demonstrated that micelles with a size below 50 nm have favorable properties, including a prolonged blood circulation and enhanced extravasation from the blood compartment into tumor tissues. This suggests that antitumor drugs packaged in micelles can be delivered to the tumor site by passive targeting to achieve accumulation at the tumor site even for tumors with low permeability [32,33,34,35].

Micelles showed zeta potential close to neutral (Table 1), which was predicted due to the presence of PEG_2k_ chains on the surface of the micelles, which, when in a water medium, forms a solvation layer in that PEG moieties are in a brush conformation. Therefore, the shear plane is sufficiently distanced from the phospholipid headgroup, which makes any possible charges on surface of nanostructures to be close to zero due to the “hidden charge effect” [36]. Although micelles have neutral zeta potential, the presence of PEG_2k_ chains on their surface can lead to steric stabilization, preventing aggregation of the system. The entrapment efficiency (EE) of PM DSPE-PEG IRN of about 88.7% indicates that the drug was well encapsulated.

### 3.2. Short-Term Stability Evaluation

**Figure 2:**
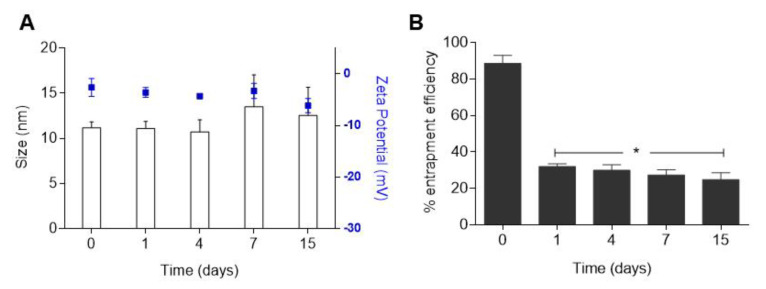
Storage stability of DSPE-PEG IRN micellar formulations stored at 4 °C for 15 days. (**A**) Mean diameter and zeta potential (blue) and (**B**) Entrapment efficiency stability over time. Note: * Represents significant differences (*p* < 0.05) compared to day 0. Data are expressed as mean ± SD (n = 3).

Stability results showed that micellar formulations remained stable within 15 days, regarding the parameters of size, size distribution, and zeta potential (Figure 2A).

The particle size of PM DSPE-PEG IRN was nearly unchanged, ranging from 10 to 15 nm (Figure 2A). D90 was consistently below 20 nm which represented homogeneity and an absence of aggregates. Zeta potential values obtained were always close to neutrality throughout the experiment. The encapsulation stability was also evaluated within 15 days and the results are shown in Figure 2B. PM DSPE-PEG showed poor encapsulation stability as a function of time, resulting in significant IRN release (~50%), after 1 day of storage (Figure 2B). Nonetheless, no significant difference in the loading amount of PM DSPE-PEG IRN from day 1 to day 15 was observed.

### 3.3. Preparation of Freeze-Dried Micelles for IRN Encapsulation

PM DSPE-PEG formulations were lyophilized and then reconstituted with IRN solution at different concentrations. The physicochemical characteristics evaluated are shown in Table 2.

As can be seen in Table 2, physicochemical characteristics of the micelles were kept as those of the non-freeze-dried micelles, indicating that the lyophilization process does not change the parameters evaluated. All samples presented sizes smaller than 20 nm and zeta potential on the micelles surface was close to neutrality that although it may indicate an incipient instability, the aggregation of micelles in solution can be avoided due to the steric hindrance promoted by the hydrophilic PEG2k chain on the surface of the nanosystem [37]. An increase in the amount of IRN encapsulated in micelles could be observed with increasing drug concentration. Based on these results, the subsequent experiments were performed using lyophilized micelles reconstituted with a 3 mg/mL IRN solution.

### 3.4. In Vitro Release Study

**Figure 3:**
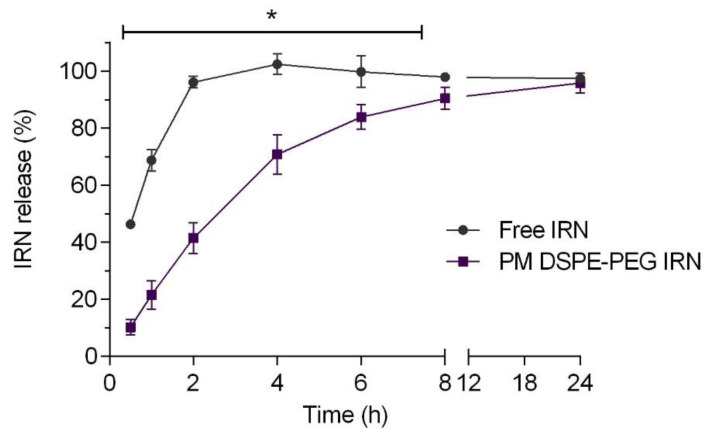
In vitro irinotecan (IRN) release profile from micellar formulations and IRN solution at 37 °C for 24 h. Note: * Represents significant differences (*p* < 0.01) between the PM DSPE-PEG IRN and Free IRN. Data are expressed as mean ± SD (n = 3).

The release of IRN from the DSPE-PEG micelles was evaluated by using the dialysis method (Figure 3). The amount of released IRN was quantified using a validated HPLC method.

Figure 3 reveals a rapid release profile for the free IRN in which about 100% of the drug was released within 2 h. In contrast, only 40% of the drug was released from DSPE-PEG micelles (PM DSPE-PEG IRN) within the same period (2 h). There was sustained release in the micelle system where it was shown to take 8 h for about 90% of the IRN releases from the micelles. These findings suggest a great potential for DSPE-PEG micellar formulations in cancer chemotherapy since minimal drug leakage before reaching the tumor site can reduce toxicity in healthy tissues and increase the safety of IRN-loaded micelles in vivo [38].

### 3.5. Hemolysis Assay

**Figure 4:**
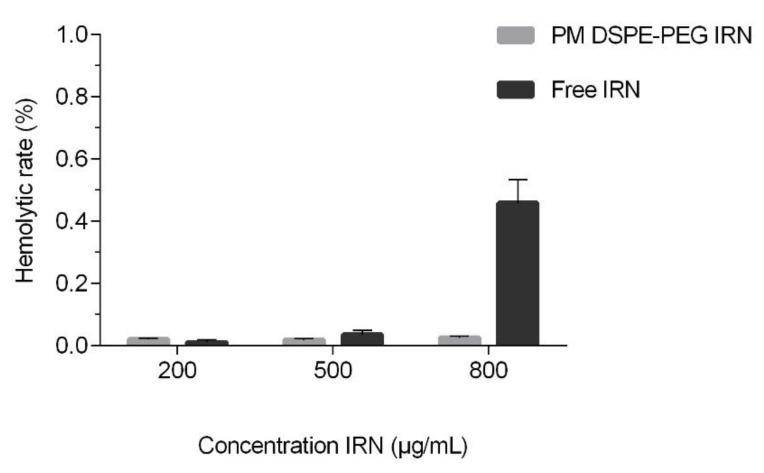
Hemolysis assay of polymeric micelles and IRN solution at various concentrations.

The hemolytic activity profiles of different concentrations of PM DSPE-PEG IRN are shown in Figure 4. It is possible to observe that the micellar formulation proposed in our study did not show significant hemolysis (<1%) of RBC at the highest concentration of 800 µg/mL or even in IRN solution. Formulations with hemolysis values lower than 2% can be considered non-hemolytic [39]. Therefore, PM DSPE-PEG IRN was shown to have hemocompatibility for intravenous administration.

### 3.6. In Vivo Antitumor Activity Evaluation

**Figure 5:**
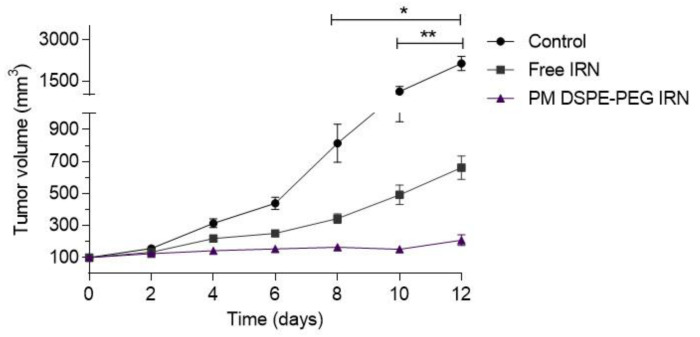
Tumor volume as a function of time after intravenous administration of saline solution (control), free IRN (IRN solution), micellar preparation IRN-loaded (PM DSPE-PEG IRN) in CT26 tumor-bearing BALB/c mice. Note: * represents a significant difference between IRN-loaded micellar treatment groups compared with the control group. ** represents a significant difference between treatments with IRN-loaded micellar formulation compared with the free IRN group (*p* < 0.05).

The antitumor activity was investigated by measuring tumor volume every other day in CT26 tumor-bearing BALB/c mice treated with saline (control), free-IRN, and PM DSPE-PEG IRN. As observed in Figure 5, mice from the control group showed a faster tumor growth due to the high rate of CT26 tumor cell proliferation. On the other hand, we observed significantly smaller tumor volumes (*p* < 0.05) in the other groups treated with IRN.

PM DSPE-PEG IRN showed to be the most effective in controlling tumor progression since the mice had the smallest tumor volumes at the end of the study. Indeed, a higher tumor inhibition ratio (Table 3) was achieved after treatments with PM DSPE-PEG IRN (88.9%) compared with free IRN (68.7%). The relative tumor volume (RTV) and inhibition rate (IR) shown in Table 3 confirm the data presented in tumor growth curves. In general, the micellar formulation was more effective in suppressing tumor growth than the free IRN or saline solution.

### 3.7. Histopathological Analysis

**Figure 6:**
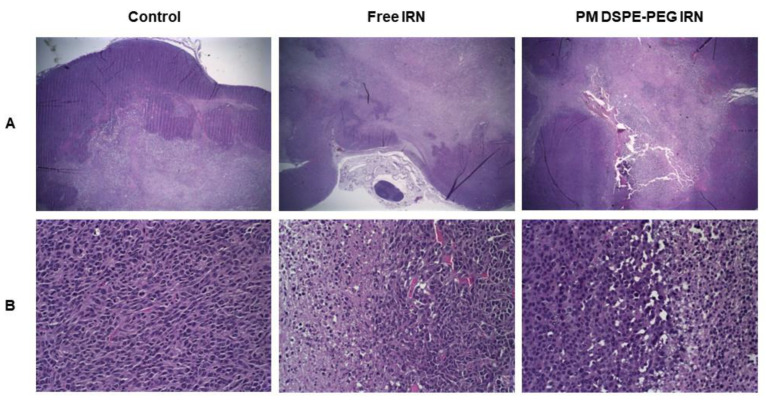
Histopathological analysis of tumor from CT26 colorectal tumor-bearing female BALB/c mice treated with saline (control), free IRN, or PM DSPE-PEG IRN stained by hematoxylin and eosin (H&E). (**A**) Original magnification 2× and (**B**) magnification 40×.

A histological study was conducted by staining tumor tissue sections with H&E (Figure 6). For all treatment and control groups were seen a solid mass with central areas of necrosis, hemorrhage, and scanty connective tissue. Most cells had pleomorphic characteristics with different nuclei shapes: round, ovoid, and spindle-shaped. Besides, the presence of mitosis indicates a high rate of tumor proliferation.

No microscopic differences could be seen in the histopathological sections between treatment groups. For tumor tissue treated with free IRN and PM DSPE-PEG IRN a transition area from necrotic tissue to viable tissue can be observed. The necrotic areas showed amorphous eosinophilic cellular debris intermixed with pyknotic nuclear debris. Similar outcomes were found in other studies disclosing large area necrosis in CT26 tumor xenografts for treated animals [26,40]. Such induction of necrosis may potentiate cancer therapy through the release of molecules that lead to cytokine production, recruitment of immune cells, and modulate dendritic cell maturation, enhancing antitumor immunity and suppressing tumor growth [41].

### 3.8. Toxicity Evaluation

**Figure 7:**
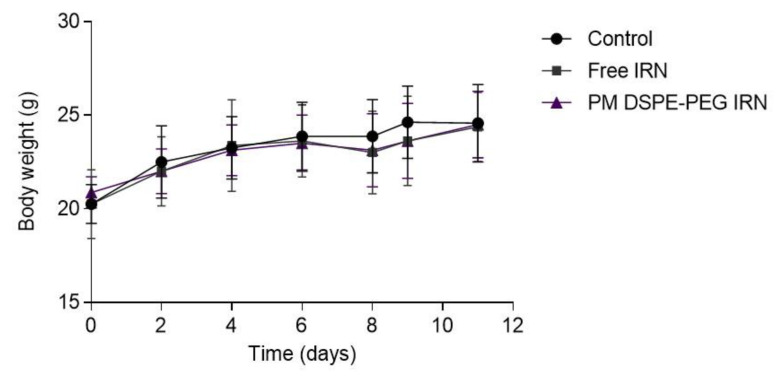
Body weight variation of treated animals. Results were expressed as the mean ± standard deviation.

Figure 7 shows that all mice receiving treatment did not present with weight loss (*p* < 0.05) and were free of morbidities, with a survival rate of 100%. The potential impact of the free drug and formulation on potential dose limiting toxicities (bone marrow, liver, and kidney) were evaluated by biochemical assays. Table 4 shows the biochemical and hematologic analyses of blood performed at the end of treatment for CT26 tumor-bearing mice treated with saline (control group), free IRN, and PM DSPE-PEG IRN. Compared to the control group, neither of the treated groups showed any alteration in kidney function as measured by urea and creatinine. The impact on liver function was evaluated by measuring ALT and AST and demonstrated that PM DSPE-PEG IRN exhibited low toxicity in vivo.

## 4. Discussion

Micelles composed of PEGylated phospholipids such as DSPE-PEG loaded with IRN at 3 mg/mL were studied the goal of improving the efficacy of IRN and mitigating its possible side effects for the treatment of colorectal cancer. The results showed that the solvent evaporation method was efficient for obtaining polymeric micelles in our study. The choice of solvent and evaporation preparation method was based on previous published studies and carried out by our research group [14,42]. This is a simple and fast method, with DSPE-mPEG_2k_ being a copolymer easily soluble in volatile solvents such as chloroform [43,44]. The lipid-polymer concentration of the DSPE-mPEG_2k_ solution in chloroform used in our study was equal to 10 mmol/L. Such concentration is about 550 times higher than the CMC value (1.8 × 10^−5^ mol/L), which was determined by ref. [42] and matches the values described in the literature as stable even after dilution in a blood volume of approximately 5 L [42,44]. The mean diameter results corroborate the values already found in the literature for DSPE-PEG_2k_ micelles [45,46,47]. Moreover, more than 90% of the synthesized micelles showed sizes smaller than 20 nm, indicating that the micellar system is homogeneous and uniform.

In general, physicochemical properties such as size, charge, and surface binders may affect the biological performance of micelles. Particles with a size less than 5 nm are rapidly cleared from the bloodstream through renal clearance while larger particles than 200 nm are more likely to undergo opsonization, recognition, and removal from circulation by the mononuclear phagocyte system (MPS) [48,49]. Furthermore, it is known that micelles with a surface charge close to neutrality can reduce the undesirable clearance by MPS, improve blood compatibility, and thus deliver the anticancer drugs more efficiently to the tumor sites by passive targeting [8,20,50]. Therefore, obtaining a small particle size around 12 nm and zeta potential near neutrality may be important to increase the blood circulation time of this system, allowing them to extravasate through the leaky vasculature and accumulate in the tumor via the enhanced permeability and retention (EPR) effect [51].

A moderate-to-high encapsulation percentage of IRN was achieved (~68%) for 3 mg/mL IRN in DSPE-PEG micelles and this finding corroborates data found by other authors that encapsulated amphiphilic drugs in lipid-core micelles [14,20,52]. Previous studies involving amphiphilic drugs such as IRN showed that the high encapsulation was associated with the drug’s ability to interact with the phosphate group present in the phosphatidylethanolamine structure and distribute at the core-shell interface of micelles [53]. Thus, the loading efficiency for different compounds correlates with the physicochemical properties of a drug as well as is associated with the interactions between drug and lipid core [54].

Although we obtained micelles with suitable IRN encapsulation, low stability of the encapsulated drug during storage was observed (Figure 2). To improve stability and allow further studies, we lyophilized the blank micelles to obtain a dry product in which the drug would be incorporated immediately before use, preventing the drug leakage. Results revealed similar physicochemical properties (e.g., size, surface charge) and IRN encapsulation compared to micelles freshly prepared, which permits in vivo antitumor activity assays [55,56].

Existing literature shows that physiochemical characteristics such as molecular weight and the compatibility between micellar core and incorporated drug influence encapsulated substance release behavior [8,57]. Therefore, the dialysis method was chosen to evaluate the IRN release profile from DSPE-PEG micelles. The results demonstrated that the release of IRN solution (free IRN) through the dialysis membrane was much faster than for drug-loaded DSPE-PEG micelles. As shown in Figure 3, the encapsulated IRN in DSPE-PEG micelles showed a controlled release behavior compared with the free drug within the first 8 h. Previous studies reported that a drug incorporated in lipid-core micelles is associated with micelles firmly enough that would slow its release [9,58]. In this case, the micelle core-forming is represented by phospholipids that, due to the hydrophobic interaction between double acyl chains, provide increased stability to micelle, which may influence the rate and release behavior of incorporated drug [59]. In contrast, other studies show that the release pattern presented can be caused by the hydrophilic moiety of the copolymer. The high polarity of PEG increases hydrophilicity and, as such, improves water solubility, causing a gradual release of the drug [60,61]. Collectively, these observations suggested that the hydrophobicity of the DSPE-PEG chains is a pivotal feature for their interaction with the drug and, consequently, impacting the release pattern.

Besides steady delivery and controlled release properties, safety is one of the most important considerations for polymeric micelles, especially for biomedical involvement as drug-delivery systems [62]. Therefore, we also investigated heme dynamics of the IRN-loaded DSPE-PEG formulation as a safety guide for intravenous administration and further in vivo application. DSPE-PEG block copolymers have been approved by the Food and Drug Administration (FDA) for medical applications and have been widely used in drug-delivery systems due to their high biocompatibility [63]. As shown in Figure 4, DSPE-PEG formulations displayed negligible hemolysis (1%) of RBCs at the highest concentration of 800 µg/mL, proving that the proposed formulation had favorable hemocompatibility and could be used as a potential nanocarrier for IRN delivery.

Antitumor efficacy was evaluated in a CT26 subcutaneous tumor mouse model. DSPE-PEG IRN micelles were effective in controlling tumor growth. On the 10th day (Figure 5), the mice receiving PM DSPE-PEG IRN formulations had lower mean RTV than mice receiving free IRN (Table 3). It is also worth noting that the groups treated with IRN-loaded micelles displayed a substantial improvement in the IR compared with the free drug (88.9% to 68.7%, respectively). This effect may be related to the increased uptake in the tumor region mediated by passive targeting and EPR effect in the tumor microenvironment [51]. Enhanced accumulation of the drugs in the tumor may have been contributed by prolonged blood circulation time and lower clearance rate [16,19]; consequently, IRN-loaded micelles caused an important tumor growth controlling profile whereas the antitumor efficacy of the free drug was modest. Additionally, our results further showed that PM DSPE-PEG IRN did not exhibit hematological or liver/kidney toxicity, and body weight changes during the whole experiment. Additionally, no obvious pathological changes in the vital organs of the mice were observed, indicating the safety of this delivery platform. Thus, the encapsulation of IRN into micelles improved in vivo antitumor efficacy with minimal toxicity, which brings convincing evidence that DSPE-PEG IRN micelles can be a promising platform for effective colorectal cancer therapy.

## 5. Conclusions

In summary, IRN-loaded DSPE-PEG micelles were successfully prepared using a simple and fast method, which showed compatible characteristics for intravenous administration. Drug release studies revealed a sustained release profile. Notably, in vivo results disclosed low toxicity for polymeric micelles, at the same time, a remarkable control in the tumor growth compared with the free IRN. Therefore, we propose that IRN-loaded DSPE-PEG micelles are an efficient and promising delivery system and hold great potential for colorectal cancer treatment.

## Figures and Tables

**Table 1:** Physicochemical properties of the different polymeric micelles prepared.

Formulation	Mean Diameter (nm) ^a^	Size Distribution ^b^	Zeta Potential (mV)	EE (%)
PM DSPE-PEG	12.4 ± 0.3	~90% < 20 nm	−2.4 ± 0.9	
PM DSPE-PEG IRN	12.6 ± 1.2	~90% < 20 nm	−2.6 ± 1.7	88.7 ± 4.4

Note: **^a^** Values expressed in terms of intensity; **^b^** Values expressed in terms of number. The results were expressed as mean ± SD (n = 3). Abbreviation: percentage entrapment efficiency (%EE).

**Table 2:** Physicochemical properties after lyophilization and reconstitution with IRN solution at different concentrations.

IRN Theoretical Concentration	Mean Diameter (nm)	Zeta Potential (mV)	Size Distribution (<20 nm)	%EE	IRN-Loading (µg/mL)
1 mg/mL	12.6 ± 1.2	−2.6 ± 1.7	97.5 ± 0.1	88.7 ± 4.4	743 ± 99
2 mg/mL	12.5 ± 2.4	0.6 ± 4.7	97.8 ± 2.8	80.2 ± 3.2	1258 ± 75
3 mg/mL	12.3 ± 1.1	−0.5 ± 0.4	99.3 ± 0.4	68.5 ± 10.5	2055 ± 382

Note: The results were expressed as the mean ± standard deviation (n = 3). Abbreviation: percentage entrapment efficiency (%EE).

**Table 3:** Relative tumor volume (RTV) and tumor growth inhibition ratio (IR) after the administration of saline (control), free IRN, and PM DSPE-PEG IRN.

Group	RTV (Mean ± SD)	IR (%)
Control	18.7 ± 9.8	
Free IRN	5.9 ± 2.0 *	68.7
PM DSPE-PEG IRN	2.1 ± 0.3 *^,#^	88.9

Note: * Represents significant difference as compared with the control group. ^#^ Represents significant difference as compared with the free IRN group. Data are expressed as mean ± SD of the mean.

**Table 4:** Hematological and biochemical parameters of CT26 tumor-bearing BALB/c mice after different treatments.

Parameters	Control	Free IRN	PM DSPE-PEG IRN
RDW (cell/mm^3^ × 10^3^)	5.3 ± 0.8	4.3 ± 1.0	5.7 ± 0.5
LYM (cell/mm^3^ × 10^3^)	1.5 ± 0.4	0.9 ± 0.2	1.2 ± 0.4
Nph (cell/mm^3^ × 10^3^)	2.7 ± 0.6	2.6 ± 0.8	2.5 ± 0.4
RBC (cell/mm^3^ × 10^6^)	6.3 ± 0.2	5.9 ± 0.4	5.7 ± 0.5
HGB (g/L)	11.7 ± 0.6	11.1 ± 0.9	10.3 ± 1.4
HTC (%)	30.6 ± 1.1	30.8 ± 1.9	28.6 ± 2.8
RDW (%)	14.7 ± 0.6	16.0 ± 1.1	14.7 ± 0.7
PLT (cell/mm^3^ × 10^3^)	380.3 ± 75.6	323. 7 ± 41.2	314.5 ± 98.0
ALT (U/L)	22.7 ± 5.5	23.3 ± 2.5	23.1 ± 3.6
AST (U/L)	146.4 ± 45.4	151. 8 ± 27.6	151.3 ± 34.5
Urea (mg/dL)	70.1 ± 7.2	83.2 ± 14.0	54.3 ± 9.6
Creatinine (mg/dL)	0.42 ± 0.10	0.39 ± 0.03	0.32 ± 0.04

Note: Results were expressed as the mean ± standard deviation. Abbreviations: RDW (total white blood cells); HGB (hemoglobin); RBC (red blood cells); HTC (hematocrit); PLT (platelets); WBC (total white blood cells); LYM (lymphocytes); Nph (neutrophils); ALT (alanine aminotransferase); AST (aspartate aminotransferase).

## Data Availability

Not applicable.
